# Effects of mobile Internet use on the health of middle-aged and older adults: evidences from China health and retirement longitudinal study

**DOI:** 10.1186/s12889-024-18916-w

**Published:** 2024-06-04

**Authors:** Ying Wang, Hong Chen

**Affiliations:** 1 School of Humanities and Management, Hunan University of Chinese Medicine, Changsha, 410208 China; 2 School of Administration and Law, Hunan Agricultural University, Changsha, 410128 China

**Keywords:** Mobile internet use, Middle-aged and elderly people, Self-rated health, Internet

## Abstract

The rapid development of digital technology has radically changed people’s lives. Simultaneously, as the population is rapidly aging, academic research is focusing on the use of Internet technology to improve middle-aged and older people’s health, particularly owing to the popularity of mobile networks, which has further increased the population’s accessibility to the Internet. However, related studies have not yet reached a consensus. Herein, empirical analysis of the influence of mobile Internet use on the subjective health and chronic disease status of individuals in their Middle Ages and above was conducted utilizing ordered logit, propensity score matching (PSM), and ordered probit models with data from the 2020 China Health and Retirement Longitudinal Study. The study aimed to provide a theoretical basis and reference for exploring technological advances to empower the development of a healthy Chinese population and to advance the process of healthy aging. The health of middle-aged and older adults mobile Internet users was greatly improved, according to our findings. Further, the use of mobile Internet by these persons resulted in improvements to both their self-assessed health and the state of their chronic diseases. As per the findings of the heterogeneity analysis, the impact of mobile Internet use was shown to be more pronounced on the well-being of middle-aged persons aged 45–60 years compared to those aged ≥ 60 years. Further, the endogeneity test revealed that the PSM model could better eliminate bias in sample selection. The results suggest that the estimates are more robust after eliminating endogeneity, and that failure to disentangle sample selectivity bias would overestimate not only the facilitating effect of mobile Internet use on the self-assessed health impacts of middle-aged and older adults, but also the ameliorating effect of mobile Internet use on the chronic diseases of middle-aged and older adults. The results of the mechanistic analysis suggest that social engagement is an important mediating mechanism between mobile Internet use and the health of middle-aged and older adults. This implies that mobile Internet use increases opportunities for social participation among middle-aged and older adults, thereby improving their health.

## Introduction

Health is a critical foundation for promoting the well-being of people and maintaining social security and stability [1, 2]. China has always given substantial importance to the construction of a medical security system and has always prioritized improving people’s health [3, 4]. Continuous efforts have considerably improved the medical level of our hair; the health level of people has also been continuously improved [5, 6]. The National Health Commission projects that by 2021, the average life expectancy in China would reach 78.2 years; this is a great improvement compared with the average lifespan in the early years of the nation was just 35 [7, 8]. Unfortunately, the rising prevalence of chronic diseases among middle-aged and older adults have been a side effect of the general increase in people’s standard of living [9, 10]. According to relevant medical survey data, the chronic disease prevalence among Chinese people aged ≥ 45 reached 75% and that among people aged ≥ 60 was as high as nearly 80%; these data suggest that the health conditions of these age groups in China are not optimistic. Simultaneously, the seventh national data suggested that 18.7% of Chinese citizens are aged 60 or older. The World Health Organization (WHO) has predicted that China would soon become a “super-aging” society. Relevant studies also show that with the increasing aging trend, the health problem of the elderly population will not be ignored, which is related to the stable development of the national economy and society [11–14]. To cope with these challenges, China has actively formulated many policies aimed at reducing middle-aged and older people’s susceptibility to developing chronic diseases and improving their health in general. The “Health China Strategy” was initially proposed in 2017 by General Secretary Xi Jinping in the report to the 19th Party Congress, which proposed to improve the national health policy, actively cope with population aging, enhance the integration of health services and revitalize the economy by improving the growth of aging businesses and industries, and offer a wide variety of medical care options to the public [15]. Since then, the State Council has implemented the “Health China 2030” planning outline, “Opinions on the Implementation of Health China Action,” and other policies to actively promote the development of China as a healthy country and improve the overall health of the nation [16, 17]. The 20th Party Congress was successfully held in 2022, during which, General Secretary Xi Jinping further emphasized his prioritization of healthy China growth, a national policy to actively manage issues related to the aging population, and an improved public health system [18, 19]. These points suggest that improving people’s health will be a long-term concern of the Party and the State for the people’s livelihood.

Continuous development of the economy and society has led to the penetration of Internet technologies represented by big data, blockchain, the Internet of things, and artificial intelligence into every aspect of residents’ lives [20–22]. Particularly, the increase in various mobile short video applications has allowed people of all ages to join the network wave [23, 24]. Based on the current figures from the 50th China Internet Development Statistics Report, the country’s Internet penetration rate was approximately 74.4% as of June 2022, and the number of unique net names in China has climbed as high as 1.051 billion. In addition, the report details that as of June 2022, there were 962 million short video users in China, an increase of 28.05 million from December 2021 and a percentage share of 91.5% of all Internet users. The above data suggest that populations in the middle-aged and older adults ranges are rapidly integrating into online society and that the era of a “universal network” has arrived. The greatest benefit of Internet technology is that it can overcome the limitations of time and space and allow interaction with information anytime and anywhere, thereby enhancing work efficiency and convenience of life in general [25]. The popularity of the mobile Internet has further enhanced its accessibility, with many people being exposed to the Internet social environment [26, 27]. The National Health Plan for the 14th Five-Year Plan was released by the State Council in 2020, and it explicitly states that the country’s citizens should leverage the Internet and related technologies to enhance the quality of their health care. Internet technology can broaden the channels for residents to search for health information so that they can search and gain health knowledge online anytime and anywhere, thereby improving their health literacy [28]. Furthermore, the use of the Internet may enhance the convenience of residents’ life, overcome the limitations of time and space, and facilitate online medical consultations and other medical behaviors [29]. As China enters a new stage of development and faces a new developmental pattern, the application of digital technology to advance the medical security system and enhance people’s health is practically significant for China to modernize the medical service system and actively and comprehensively promote healthy aging and the “Health China Strategy.”

Based on this background, the present study aimed to use data from the 2020 China Health and Aging Tracking Survey (CHARLS) to empirically examine the influence that using mobile Internet has on the health of persons in their middle age and later years. The study results not only encourage a new academic perspective and thinking for studying health policies for the elderly population in the context of technological progress but also provide new empirical evidence for government departments to develop health policies in the era of the digital network. The remaining chapters of this study have been organized as follows: the second section is the literature review, in which existing literature has been reviewed and summarized; the third section highlights the data and methods, including data sources, variable design, and model construction; the fourth section summarizes the study results, with statistical results of baseline regression, robustness test, heterogeneity analysis, and endogeneity elimination; the fifth section is the discussion section, in which the results of this study have been discussed in detail and the strengths and limitations have been stated; the last section is the conclusion of this study.

## Literature review

With the continuous development of digital technology, there has been a surge in the number of research reports that investigate the connection between breakthroughs in technology and improvements in public health. The present study compared and summarized the findings of relevant studies and observed that the majority of contemporary research has been on analyzing how conventional Internet use affects the health of populations and that the findings of the research that investigated the connection between people’s use of the Internet and their overall health mostly focused on two aspects: Internet use and citizens’ health have a strong favorable association, i.e., Internet use improves residents’ health, and that Internet use is inversely correlated with citizens’ health., i.e., the Internet is detrimental to residents’ health.

The first conclusion suggests that Internet use may improve population health. Wang et al. analyzed the influence of Internet use on the health of senior individuals using data from the 2012 and 2015 China General Social Survey (CGSS), and they discovered that Internet use significantly affected the users’ physical and mental health, but had no significant influence on self-reported health [30]. The connection between Internet use and psychological and physiological health was also moderated negatively by the user’s cognitive abilities. Using data from the 2018 CHARLS, Li et al. conducted an empirical study to determine whether or not frequent Internet use negatively impacts the health of Chinese adults aged 40 and above. They found that adults in their middle years and beyond who used the Internet had better estimates of their health and were less likely to develop chronic disorders [31]. Nonetheless, Internet use had a greater impact on boosting the status of chronic diseases than on users’ perceptions of their health. Using data from the 2017 CGSS, Han and Zhao categorized overall health into social, mental, and physical health and studied the effects of Internet use on multidimensional health using the ordered probability model. As per their findings, using the Internet regularly may benefit health in a variety of ways [32]. Liu et al. reported that self-reported health was better among Chinese seniors who utilized the Internet, and seniors who reported high levels of social support from family and friends also reported much better health [33]. According to Guo et al., depressive symptoms in the elderly were shown to be decreased by 3.370 points, or around 37.19%, when Internet use was compared to non-Internet use. The health effects were particularly prominent for agricultural workers, women, and older adults [34]. A double-difference approach was used by Fan and Yang, who analyzed CHARLS panel data from 2013, 2015, and 2018, to determine whether or not Internet use negatively impacted the mental health of those in their middle ages and beyond in rural China and reported that the participants’ mental health greatly benefited from their Internet use [35]. Guo et al. used data from 2014, 2016, 2018, and 2020 Chinese Household Panel Surveys to study Internet use and its consequences on the health of the elderly. They found that Internet use has a considerable impact on the physical health of older adults, particularly women, rural residents, and the Midwest population [36].

The second finding is that excessive use of the Internet has a detrimental impact on the overall health of individuals. Chen et al. found that compared with females, males are more likely to be addicted to Internet gaming, leading to Internet addiction, which, in turn, affects their health [37]. Additionally, Kwak found that residents who spent more time online than their peers reported having lower subjective health, greater stress levels, and more intense emotions of despair and suicidal thoughts than those who spent less time online [38]. Cai et al. reported that concerns regarding the negative impacts of excessive Internet use on users’ psychological health have grown as its use has increased. They discovered that excessive online activity was marginally linked to symptoms of depression, anxiety, solitude, and other psychological health problems, and was adversely linked to subjective well-being [39]. A study by Xie et al. found that Internet use affects the mental health of older adults and increases their prevalence of depressive symptoms. Further heterogeneity analyses showed more pronounced negative impacts on mental health for specific groups of older persons, such as females, young and middle-aged, high income, non-rural, less educated, and older persons living with others [40]. Zhang et al. used data from the 2018 Chinese Family Panel Studies (CFPS2018) to assess the impact of Internet use on the mental health of 14,497 middle-aged and elderly people. The findings suggest that excessive Internet use can lead to increased levels of depression and decreased cognitive function [41].

In terms of Internet use and social participation, studies have shown that Internet use can significantly enhance the social participation of the middle-aged and elderly population. Gong et al. analyzed the impact of Internet use on the social participation of the elderly using data from the China Longitudinal Aging Social Survey (CLASS) 2018, and found that Internet use has expanded the areas of social participation of the elderly in China [42]. Using data based on the 2018 China Family Panel Studies, Dong et al. empirically analyzed the impact of Internet use on the social participation of urban older adults. The study found that Internet use has a significant positive impact on political participation and voluntary participation of urban older adults [43]. In terms of social participation and health, studies have shown that social participation can significantly improve the health status of middle-aged and elderly people. Liang et al. used the data from the 2018 Chinese Longitudinal Healthy Longevity Survey (CLHLS) to comprehensively analyze the impact of social participation on the health of the elderly using logistic regression modeling and propensity score matching method, and found that social participation can improve the physical and mental health of the elderly [44]. He et al. argues that social participation is an important action program to promote mental health in old age and realize active aging. They analyzed the impact of social participation models on the mental health of older adults using data from the 2015 and 2018 China Health and Retirement Longitudinal Study. The study found that different modes of social participation were able to improve the mental health of older adults [45].

In summary, studies have drawn two main conclusions about the correlations between Internet use and people’s health; nonetheless, no agreement has been achieved. As China advances into a new stage of development, demographic structure, socioeconomic environment, and social development pattern have undergone drastic changes. Therefore, it is practically important to revisit and discuss the correlation of technological advances with population health to realize the strategy of a healthy China, alleviate the problems caused by an aging society, promote the health and well-being of the population, and comprehensively promote the modernization of the national health care system. Past research has mostly concentrated on conventional Internet use; however, the primary objective of this research was to investigate the implications that using mobile Internet has on the health of populations; this is particularly important for exploring the health effects of mobile Internet in a comprehensive network era. Second, previous studies mostly focused on the entire population, elderly people aged ≥ 60, and adolescents under the age of 18; research including both middle-aged and older adults (aged ≥ 45) as subjects are lacking. In addition, fewer studies have analyzed social engagement as a mechanism of action between Internet use and the health of middle-aged and older adults. With the increase in average life expectancy and changes in population outcome, individuals in the middle age range and those in their later years become the main workforce for our social and economic development. Meanwhile, as the government’s policy of delaying retirement age will soon be implemented, re-employment of elderly people will become the main concern of the government in terms of future social security. Therefore, investigating the connection between the use of technology and the health of adults in their middle years and above is of the utmost importance to enhance the healthcare system and improve their quality of life. In terms of research methodology, previous research has mostly ignored the issue of endogeneity. However, to better quantify the potential risks of mobile Internet use on users’ health, we employed the propensity score matching (PSM) model to control for bias in sample selection.

By using information from the 2020 CHARLS, this research sought to empirically examine the influence of mobile Internet use on the health of individuals within the middle age range and older adults. Robustness tests and heterogeneity analysis were conducted and the PSM model was used to determine the overall impact that using mobile Internet has on the health of persons in their middle age and later years, thereby eliminating the issue of endogeneity due to sample selectivity bias.

## Data and methods

### Data sources

The CHARLS 2020 database served as the source for the information presented in this article. It is a large-scale project hosted by Peking University’s Institute of Development Studies and jointly implemented by Peking University’s China Social Science Survey Center and Peking University’s Youth League Committee. The major aim of this project is to collect high-quality micro-survey data on Chinese middle-aged and older households and individuals aged ≥ 45. In 2011, a nationwide baseline survey was carried out; There were a total of 450 localities (villages) and 150 counties surveyed throughout 28 provinces (incorporated municipal authorities and autonomous areas reporting directly to the Central Government) in 2011, 2013, 2015, 2018 and 2020; this encompasses a wide range of data and has a strong representation in China. In our study, we used the latest 2020 database. Because the research topic of this study is the impact of Internet use on health, the individual pool was selected for analysis. After data processing, censoring, and elimination of invalid variables, a final valid sample of 8491 was acquired.

### Variable design

#### Dependent variable

The health of individuals in their middle years and beyond served as the study’s dependent variable. To comprehensively measure this variable, similar to previous studies, the health of the study population was evaluated by combining self-reported well-being with the presence or absence of chronic disease [46, 47]. In the 2020 CHARLS questionnaire, for the question “How do you think your health is? Is it very good, good, fair, bad or very bad,” we allotted a scale of 1 to very bad, 2 to bad, 3 to fair, 4 to good, and 5 to very good. The quantiles of 25%, 50%, 75% of self-rated health were classified as 2, 3, 4. The questionnaire listed 15 chronic diseases, specifically, stomach or digestive system disease, kidney disease, stroke, liver disease, chronic lung disease, heart disease, cancer (malignancy), diabetes or elevated blood glucose, dyslipidemia (high or low blood lipids), hypertension, emotional and mental disease, dementia, parkinson, arthritis or rheumatism, and asthma. If the responder possessed any of these conditions, a score of 1 was allotted, signifying that they have a chronic disease; otherwise, the default score of 0 was set, signifying that they do not have a chronic disease.

#### Independent variable

Mobile Internet use was used as the independent variable. The questionnaire included the following question: “Which of the following tools do you use to access the Internet?” If the respondent chose to use a tablet or mobile to access the Internet, the value assigned was 1, implying that they are active users of the mobile web; otherwise, the value assigned was 0, implying that they are not mobile Internet users. In order to further portray mobile Internet usage, we utilize the number of mobile Internet features used by middle-aged and older adults as a proxy variable, which is used to conduct robustness tests. The health of middle-aged and elderly people is also influenced by personal characteristics, such as gender, age, education level; Lifestyle, such as smoking, alcohol and other factors. Drawing on related studies [48, 49], the variables respondents’ sex, age, marital status, education level, residential address (rural/urban), employment status, health insurance participation, and lifestyle variables (e.g., smoking, alcohol consumption, etc.) were also included in the model as control variables for analysis. In the 2020 CHARLS database, the question “What is your gender?” Female = 0, Male = 1. “What is your marital status?” Married = 1, separated = 2, divorced = 3, widowed = 4, unmarried = 5. “What is your education level?” Illiterate = 1, primary = 2, secondary = 3, university and above = 4. “What is your residential address?” urban = 1, urban-rural = 2, rural = 3. “Are you currently employed? No = 0, Yes = 1.“Do you participate in medical insurance? No = 0, Yes = 1. “Do you smoke?” No = 0, Yes = 1. “Do you drink alcohol?” No = 0, Yes = 1. The assignment of variables and descriptive statistics findings are displayed in Table 1.

#### Intermediate variable

Social participation is the mediating variable in this paper. In order to further analyze the channel of the mechanism of action of the impact of mobile Internet use on the health of middle-aged and elderly people. In this paper, we choose the social participation variable for the mediation test. In the questionnaire, there is a question “Did you do any social activities in the past month?” The answer options include: visiting the door to socialize with friends, playing mahjong or chess, providing help to others, dancing or working out, participating in club activities, participating in volunteer activities, attending training courses, and others. If the respondent participates in any of the above social activities, he/she is considered to have engaged in social participation, which is replicated as 1. Otherwise, a value of 0 is assigned.

**Table 1:** Variable assignment and descriptive statistics results

Variable name	Variable definition	*N*	Mean	SD	Min	Max
Dependent variable						
Self-rated health	Very bad = 1, bad = 2, fair = 3, good = 4, very good = 5	8491	3.664	0.764	1	5
Chronic diseases	No = 0, Yes = 1	8491	0.793	0.405	0	1
Independent variable						
Mobile Internet use	No = 0, Yes = 1	8491	0.439	0.496	0	1
Control variables						
Sex	Female = 0, Male = 1	8491	0.214	0.410	0	1
Age	Unit: years	8491	64.741	9.398	45	102
Marital status	Married = 1, separated = 2, divorced = 3, widowed = 4, unmarried = 5	8491	1.289	0.700	1	5
Education level	Illiterate = 1, primary = 2, secondary = 3, university and above = 4	8491	2.115	0.798	1	4
Residential address	Urban = 1, urban-rural = 2, rural = 3	8491	2.380	0.856	1	3
Employment status	No = 0, Yes = 1	8491	0.493	0.500	0	1
Medical insurance	No = 0, Yes = 1	8491	0.957	0.203	0	1
Smoking	No = 0, Yes = 1	8491	0.068	0.252	0	1
Drinking	No = 0, Yes = 1	8491	0.254	0.435	0	1
Sleep time	Unit: hour	8491	6.387	1.529	4	15
Intermediary variable						
Social participation	No = 0, Yes = 1	8491	0.506	0.500	0	1

### Model design

Empirical research on the topic of the health of older adults was carried out using the ordered logit and binary logit models because the explanatory factors consisted of five categorical and dichotomous variables. The specific econometric model is as follows:

1 $${\rm{Health}}\,{\rm{ = }}\,{\rm{ln}}\left( {{{\rm{P}} \over {{\rm{1 - P}}}}} \right)\,{\rm{ = }}\,{{\rm{\alpha }}_{\rm{0}}}\,{\rm{ + }}\,{{\rm{\alpha }}_{\rm{1}}}{\rm{internet}}\,{\rm{ + }}\,{\rm{\beta X}}\,{\rm{ + }}\,{\rm{\varepsilon Internet}}$$

In the above equation, Health denotes the explanatory variable, i.e., middle-aged and elderly people’s health conditions, Internet denotes the explanatory variable Internet use, ${\rm{\beta }}$ denotes the estimated coefficients, X denotes the control variables, and ${\rm{\varepsilon }}$ denotes the random perturbation term. To ensure the robustness of the analysis, we used the ordered probit and probit models as replacement econometric models. The specific econometric models are as follows:

2 $${\rm{Healt}}{{\rm{h}}_{\rm{i}}}{\rm{ = \alpha + }}{{\rm{\beta }}_{\rm{1}}}{\rm{interne}}{{\rm{t}}_{\rm{i}}}{\rm{ + \theta }}{{\rm{X}}_{\rm{i}}}{\rm{ + }}{\varepsilon _{\rm{i}}}{\rm{Internet}}$$

In Eq. (2), ${\text{Health}}_{\text{i}}$ indicates the state of health for those in their middle and later years, Internet denotes whether the individual uses mobile Internet, ${\text{X}}_{\text{i}}$ denotes the control variables, and ${{\rm{\varepsilon }}_{\rm{i}}}$ is the random perturbation term. ${{\rm{\beta }}_{\rm{i}}}$ is the estimated coefficient of the model. Because mobile Internet use belongs to sample self-selection behavior, the model results will have the issue of endogeneity; therefore, to avoid the presence of endogeneity, we employed the PSM model [50, 51]. The econometric model is as follows:

3 $${{\rm{Q}}_{\rm{i}}}\,{\rm{ = }}\,{{\rm{Q}}_{{\rm{0i}}}}{\rm{ + }}\left( {{{\rm{Q}}_{{\rm{1i}}}}{\rm{ - }}{{\rm{Q}}_{{\rm{0i}}}}} \right){{\rm{Z}}_{\rm{i}}}$$

4 $${\rm{ATT}}\,{\rm{ = }}\,{\rm{E}}\left( {{{\rm{Q}}_{{\rm{1i}}}}{\rm{ - }}\left. {{{\rm{Q}}_{{\rm{0i}}}}} \right|{{\rm{Z}}_{\rm{i}}}{\rm{ = 1}}} \right)$$

In Eq. (3), ${\text{Z}}_{\text{i}}$ denotes the treatment variable; If the score is 1, then individual i is part of the study’s experimental group, and if the score is 0, it indicates that the individual is in the control group. Equation (4) denotes the average treatment effect on the treated (ATT), which is the impact that using mobile Internet has on the health of persons in their middle years and older after eliminating endogeneity.

## Results

### Baseline regression outcomes

Models (1) and (2) show the baseline regression outcomes of the influence of using mobile Internet on people’s perceptions of their health, specifically those of middle-aged and elderly individuals. Models (3) and (4) show the findings on the impact that using mobile Internet has on the prevalence of chronic diseases across individuals of middle age and older. According to the findings, using mobile Internet has a substantial impact on both self-reported health and the prevalence of chronic diseases among persons of middle age and older. Concerning self-rated health, model (1) displays the regression findings in the absence of control variables, while model (2) displays the regression findings with control factors included. Both models indicate that mobile use of the Internet has the potential to substantially enhance populations’ health, which means that self-reported health is better among those in their middle age and above who use mobile Internet compared to those of the same demographic who do not. In terms of chronic disease prevalence, both models (3) and (4) indicate that the health of those in their middle years and senior citizens may be greatly enhanced by their Internet use. Thus, there is a link between mobile web activity and a decreased risk of chronic disease in middle-aged and elderly persons. Therefore, mobile Internet positively contributes to the health condition of those in their middle and later years.

The findings for the control variable generally matched those of earlier research. When comparing the sexes, males had a high-self rating score on their health than women. Generally, middle-aged and older adults health deteriorates as they age, and the chances of suffering from chronic diseases are higher, which is consistent with the current social situation in China [52, 53]. As far as marital status is concerned, the unmarried group is significantly healthier than the married going group. In terms of education levels, adults who were more educated showed worse self-rated health statuses; this may be because highly educated individuals in the middle age range and above can make more objective judgments about their health status. The results show that people who drink alcohol tend to have worse self-rated health, but the results of this study show that drinking alcohol does not adversely affect chronic disease. This result may be due to the heterogeneity of drinking behavior in the samples of this study, for example, individuals with different levels of education often have large differences in drinking rating. As we all know, many parties such as the World Health Organization have shown that alcohol is an important influence in causing chronic diseases, especially leading to malignant tumors [54–56]. Sleep duration was positively linked to better subjective and objective measures of health, including those for chronic disease, indicating that middle-aged and elderly adults with adequate sleep duration tended to have good overall health. According to the results of descriptive statistics, the mean value of sleep time of Chinese middle-aged and elderly people is 6.387, which indicates that Chinese middle-aged and elderly people have enough sleep time, and therefore the better their health condition. Table 2 displays the findings of the baseline regression.

**Table 2:** Baseline regression results

Variables	Model (1)	Model (2)	Model (3)	Model (4)
Mobile Internet use	0.190*** (0.042)	0.128** (0.053)	−0.422*** (0.054)	−0.090** (0.068)
Gender		0.257*** (0.062)		−0.207*** (0.080)
Age		−0.0105** (0.003)		0.061*** (0.004)
Marital status		0.068** (0.032)		−0.013 (0.047)
Education levels		−0.137*** (0.032)		−0.024 (0.042)
Residential address		0.099*** (0.029)		-0.003 (0.038)
Employment status		−0.221*** (0.047)		0.109* (0.063)
Medical insurance		−0.013 (0.103)		0.239* (0.133)
Smoking		−0.026 (0.092)		0.110 (0.120)
Drinking		−0.158*** (0.053)		−0.131* (0.068)
Sleep duration		0.053*** (0.014)		−0.152*** (0.018)
N	8491	8491	8491	8491
Ps-R^2^	0.0012	0.0067	0.0071	0.0525

Note *, **, and *** denote significance at 10%, 5%, and 1% levels, correspondingly

### Analyses of robustness

In order to ensure the robustness of the results obtained and to further characterize the impact of mobile Internet use on the health of middle-aged and elderly people, this paper utilizes the substitution of measures and the substitution of independent variables for the robustness test. For the multicategorical variable of self-assessed health, this paper replaces the ordered logit model with the Order Probit model; for the dichotomous variable of chronic disease status, this paper replaces the Logit model with the Probit model. In terms of replacing the independent variables, this paper utilizes the number of mobile Internet function use to replace mobile Internet use for the robustness test. Table 3 shows the results of the robustness test. Models (5) and (6) are the results of the analysis of the replacement econometric model, and models (7) and (8) are the results of replacing the independent variables. According to the results, mobile Internet use has a significant positive impact on self-assessed health and chronic disease status among middle-aged and older adults, both with the replacement of the measurement model and the replacement of the core independent variables. This result is consistent with the benchmark results, indicating that the conclusions obtained in this paper are robust.

**Table 3:** Results of robustness analysis

Variable Name	Model (5)	Model (6)	Model (7)	Model (8)
Mobile Internet use	0.081** (0.032)	−0.049** (0.039)		
Mobile Internet functions used			0.055*** (0.020)	−0.005** (0.025)
Sex	0.161*** (0.037)	−0.124*** (0.046)	0.253*** (0.062)	−0.209*** (0.080)
Age	−0.007*** (0.002)	0.034*** (0.002)	−0.010*** (0.003)	0.059*** (0.004)
Marital status	0.041** (0.019)	−0,011 (0.026)	0.067** (0.032)	−0.013 (0.047)
Education levels	−0.086*** (0.019)	−0.010 (0.024)	−0.134*** (0.032)	-0.009 (0.042)
Residential address	0.058*** (0.018)	0.002 (0.022)	0.097*** (0.029)	−0.007 (0.038)
Employment status	−0.134*** (0.029)	0.053 (0.036)	−0.222*** (0.047)	0.105* (0.063)
Medical insurance	−0.016 (0.062)	0.151** (0.076)	-0.016 (0.103)	0.244* (0.133)
Smoking	−0.012 (0.055)	0.068 (0.069)	-0.025 (0.092)	0.111 (0.120)
Drinking	−0.094*** (0.032)	−0.080** (0.039)	−0.154*** (0.053)	−0.125* (0.068)
Sleep duration	0.034*** (0.008)	−0.089*** (0.011)	0.052*** (0.020)	−0.153*** (0.018)
N	8491	8491	8491	8491
Ps-R^2^	0.0070	0.0516	0.0068	0.0523

Note *, **, and *** denote significance at 10%, 5%, and 1% levels, correspondingly

### Analyses of heterogeneity

To further analyze the influence of using mobile Internet on the health status of individuals in the middle age range and above, we examined heterogeneity from the perspectives of different ages. Adults were categorized as either middle-aged (45–60 years old) or elderly (60 + years old) based on the WHO’s definitions. The heterogeneity analyses revealed significant heterogeneity in both subjective health and chronic disease status. In terms of subjective health, mobile Internet use was meaningfully correlated for the middle-aged group but was not correlated for the elderly group. In terms of chronic disease status, mobile Internet use improved the status of chronic disease individuals in their middle ages but did not improve that of the elderly group. Table 4 presents the findings from the heterogeneity analyses.

**Table 4:** Heterogeneity analyses

	Self-rated health		Chronic disease conditions	
	45 ≤ Age <60	Age ≥ 60	45 ≤ Age <60	Age ≥ 60
Mobile Internet use	0.135** (0.077)	0.267 (0.056)	−0.007** (0.086)	−0.081 (0.082)
Control variables	YES	YES	YES	YES
N	2955	5536	2955	5536
Ps-R^2^	0.0005	0.0020	0.0102	0.0002

### Endogenous elimination

**Fig. 1:**
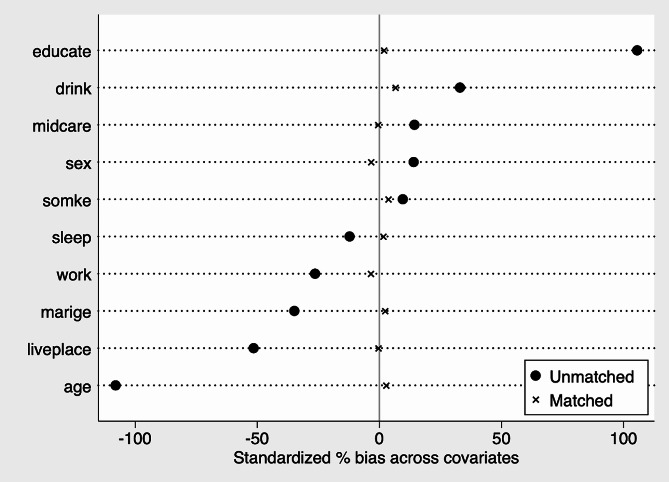
Equilibrium chart

Sample selection bias might lead to endogeneity issues in the empirical findings since mobile Internet use is a freely chosen behavior. Related studies have shown that various education levels, marital status, and lifestyles can affect the mobile Internet use of residents [57, 58]. We employed a PSM model to get rid of the endogeneity issues arising from bias in sample selection. PSM model dictates that a sample balance test comes first in the process, and we can enter the subsequent analysis only when the sample passes the balance test [59]. Table 5 shows that before matching, all factors were statistically significant in the balance test, whereas all of them were insignificant after matching, indicating that the matched sample had improved balance. Furthermore, the balance test plot of Fig. 1 with variable sub-balance indicates that the matched samples are well balanced. Table 5 displays the specific results of each balance test, and Fig. 1 depicts the corresponding balance test plots.

**Table 5:** Sample matching quality balance test

Variables	Matching Situation	Mean		Bias (%)	Reduce Bias (%)	t-Test	
		Treated	Control			t-value	*P* > |t|
Sex	Before	0.245	0.188	14.0	75.5	6.45	0.000
	After	0.244	0.259	−3.4		−1.40	0.161
Age	Before	59.793	68.638	−108.0	97.5	−48.67	0.000
	After	59.868	59.645	2.7		1.40	0.163
Marital status	Before	1.156	1.391	−34.8	93.5	−15.56	0.000
	After	1.157	1.142	2.3		1.30	0.195
Education levels	Before	2.533	1.787	105.6	98.3	48.19	0.000
	After	2.521	2.508	1.8		0.81	0.416
Residential address	Before After	2.138 2.147	2.570 2.151	-51.5 -0.5	99.1	-23.84 -0.18	0.000 0.856
Employment status	Before After	0.419 0.422	0.549 0.439	-26.4 -3.6	86.5	-12.04 -1.53	0.000 0.126
Medical insurance	Before After	0.972 0.972	0.944 0.973	14.3 -0.6	95.8	6.43 -0.32	0.000 0.749
Smoking	Before	0.081	0.057	9.6	62.2	4.42	0.000
	After	0.080	0.071	3.6		1.50	0.134
Drinking	Before	0.334	0.191	33.0	80.2	15.26	0.000
	After	0.330	0.301	6.5		2.62	0.109
Sleep duration	Before	6.283	6.467	−12.2	87.3	−5.50	0.000
	After	6.282	6.259	1.6		0.74	0.459

*Note* Treated indicates the processing group; Control indicates the control group

The outcomes of the PSM analysis, i.e., the ATT, are displayed in Table 6. The analysis in this research included the use of both K-nearest neighbor and kernel matching to ensure the reliability of the findings. In terms of self-rated health, the pre-match ATT value was 0.0748 and the post-match values were 0.0469 and 0.0571, respectively, suggesting that the impacts of mobile web activity on the subjective health of individuals in the middle age range and above are overstated if endogeneity is not eliminated. In terms of chronic disease status, the pre-match ATT value was − 0.0699 and the post-match values were − 0.0263 and − 0.0165, respectively, indicating a decrease in the absolute values. Thus, if endogeneity is not accounted for, the beneficial benefits of mobile Internet use on middle-aged and older adults with chronic diseases would be overstated Table 6 depicts the findings of the targeted ATT estimate. Table 6 depicts the findings of the targeted ATT estimate.

**Table 6:** Estimated results of the average treatment effect

	Self-rated health				Chronic disease conditions			
	Treated	Control	ATT	SE	Treated	Control	ATT	SE
Before	3.6215	3.6964	0.0748	0.0166	0.7536	0.8236	−0.0699	0.0088
After								
K-Nearest Neighbor matching	3.6205	3.6675	0.0469	0.0284	0.7543	0.7279	−0.0263	0.0176
Kernel matching	3.6205	3.6196	0.0571	0.0282	0.7543	0.7377	−0.0165	0.0144

*Note* The “one-to-four” matching method was employed by K-nearest neighbor matching, and Kernel matching was performed with the parameters of kernel function and bandwidth left at their default levels

### Mechanism analysis

In order to further analyze in depth the relationship between mobile Internet use and the self-assessed health and chronic disease status of middle-aged and elderly people, this paper explores the mechanism of action between mobile Internet use and the self-assessed health and chronic disease status of middle-aged and elderly people by using the mediation effect model. Relevant studies have shown that with the development of digital technology, on the one hand, Internet use can increase the opportunity and frequency of residents’ social participation, and on the other hand, social participation can significantly improve residents’ health status [60, 61]. To verify its mediating effect, this paper selects social participation as a mediating variable to verify its intrinsic mechanism between mobile Internet use and middle-aged and elderly people’s self-assessed health and chronic disease status. We draw on the basic idea of Baron [62] on mediating effect analysis and utilize the stepwise regression method for mediating effect analysis. According to the results in Table 7, at stepwise regression, mobile Internet use has a significant positive effect on both the health status and social participation of middle-aged and older adults, respectively. This result still holds when these two variables are included together in the model for analysis. This result suggests that social participation has a significant and partially mediated effect between mobile Internet use and the health of middle-aged and older adults. The specific pathway is that mobile Internet use increases opportunities for social participation among rural middle-aged and older adults, which improves their health. The results of the mediation effect estimates are shown in Table 7.

**Table 7:** Estimates of the mediating effect of social participation

Step	Self-rated health			Chronic disease conditions		
	Step 1	Step 2	Step 3	Step 1	Step 2	Step 3
Variable	Self-rated health	Social participation	Self-rated health	Chronic disease conditions	Social participation	Chronic disease conditions
Mobile Internet use	0.128** (0.053)	0.383*** (0.034)	0.073** (0.032)	−0.090** (0.068)	0.383*** (0.034)	-0.038* (0.039)
Social participation			0.045** (0.025)			-0.072* (0.032)
Control variable	YES	YES	YES	YES	YES	YES
Adj-R^2^	0.0067	0.0359	0.0072	0.0525	0.0359	0.0522
N	8491	8491	8491	8491	8491	8491

## Discussion

### The use of mobile internet may considerably enhance older individuals’ health

The findings of this investigation corroborate the premise that persons of the middle age range and above may benefit greatly from using mobile Internet to strengthen their self-evaluated health and reduce their risk of developing chronic diseases. These findings are in line with what Li and Wang and colleagues found in their research [30, 63]. Data shows that China’s 5G base stations account for > 60% of the global total, proving the success of China’s recent efforts to support the building of a digital China. This has laid a solid foundation for enhancing the Internet penetration rate of residents. Notably, With the advent of mobile Internet, it is now possible for people to obtain medical advice from specialists at any time and from any location, considerably improving the quality of care they receive [64]. In particular for individuals of middle age and above, mobile Internet use can effectively streamline mobility-related problems, enabling them to provide appropriate medical services promptly, and this can improve their health conditions [65]. On the other hand, mobile Internet eliminates information asymmetry and widens information access. Through daily observations, it is easy to understand that many middle-aged and elderly adults will learn about fitness and health through the Internet, which will remarkably improve their health-related literacy and subsequently, their health levels [28]. In summary, relevant departments should pay attention to the health-promoting effects of the Internet on older people and those in their middle years. Additionally, the departments can actively guide people in their late middle years and beyond to efficiently use the Internet and improve their mobile Internet use skills, maximizing the health benefits of mobile Internet. Furthermore, relevant departments should also note that the phenomenon of the “digital divide” continues to exist and certain middle-aged and elderly adults should not be exposed to the Internet. Thus, there is an urgent need to develop mechanisms to break the “digital divide” so that a large proportion of the adult population, including those of middle and old age can avail the dividends of the digital age.

### Heterogeneity of the effects of mobile Internet use on the health of elderly and middle-aged persons in different age groups

The findings illustrate that mobile Internet use is highly heterogeneous across age groups of individuals in the middle age range and above. In particular, the health-promoting effects of mobile Internet were more pronounced among the middle-aged group of individuals aged 45–60 years than those aged ≥ 60 years. This may be attributed to the improved Internet use skills of middle-aged individuals, whereas the older age group had relatively lower odds and skills of using the Internet [66]. Overall, the middle-aged population was slightly better than the elderly population in terms of economic strength and education, and this population was not only proficient in utilizing the web for health-related research and education, but also for “Internet medical” and “Internet hospitals,” both of which may substantially enhance the quality of their health [67, 68]. In contrast, elderly individuals are often excluded from Internet use due to the social environments of the current times and the degradation of their physical and mental functions. Even when they use the Internet, their skills are very weak and particularly weak when using the Internet to promote their health [69–71]. Therefore, in the future, studies should focus on elderly individuals aged ≥ 60 years to strengthen their social integration and enable them to be integrated into the universal Internet society. Furthermore, we should adopt various channels to increase the training of Internet skills for the elderly; for example, the community as a unit can be explained Internet-related knowledge and use skills through regular lectures or peer-to-peer visits, so that they can overcome the fear of using the Internet as elderly adults and improve their Internet use skills during daily life.

### PSM can effectively eliminate the issue of endogeneity

Owing to differences in endowment resources, there will be differences in Internet use behavior amongst individuals in the middle age range and up with different education levels, marital status, or lifestyles; this can lead to the issue of endogeneity in model estimation results owing to bias in the selection of samples. Unless this endogeneity is taken into account, the model estimation results may be inaccurate, and the health-promoting effects of online activity on individuals of middle age and beyond will be overestimated or underestimated. Nevertheless, most previous studies have tended to ignore this endogeneity problem. In the present study, we eliminated the issue of endogeneity by using the PSM model. We observed that if the endogeneity issue was not addressed, the positive impact of mobile Internet use on the perceived health benefits of middle-aged and older adults is overestimated, and the beneficial impact of mobile Internet on the improvement of chronic disease in adults over middle age is exaggerated. This indicates that the PSM model better eliminates endogeneity and that the obtained estimation results have better scientific validity [72]. The estimated findings were made reliable by the application of the replacement econometric model. We observed that the estimated values are quite reliable and that the conclusions obtained are scientifically sound and highly credible.

### Social participation as an important mechanistic channel between mobile Internet use and the health of middle-aged and older adults

The findings of this paper suggest that social participation is an important mechanism channel between mobile Internet use and the health of middle-aged and older adults. This means that mobile Internet use increases the opportunities for social participation of middle-aged and elderly people, which in turn enhances their health. With the continuous popularization of the Internet, middle-aged and elderly people can use the mobile Internet platform for social chatting, entertainment, shopping, etc., which greatly enhances the opportunities for social participation of middle-aged and elderly people. On the one hand, the Internet can break through the limitations of time and space, allowing the elderly to contact friends and family members anytime and anywhere, thus enhancing the relationship between the elderly and social communication and interaction, and thus enabling them to maintain their physical and mental well-being. On the other hand, mobile Internet technology can eliminate the asymmetry of information and enable the elderly to know more social information, thus increasing their social participation and improving their health. Therefore, the government should not only consider strengthening the popularization and promotion of mobile Internet among the elderly in its future policy formulation, providing them with more convenient platforms for information acquisition and communication, but also encouraging the elderly to actively participate in social activities, expanding the scope of social interaction and enhancing social support.

### Innovations and limitations

Data from the 2020 CHARLS was used in conducting an empirical analysis of the influence of mobile Internet use on the health of Chinese adults of middle age and above utilizing the ologit and PSM models. This has important policy implications for fostering a healthy China, easing the burden of an aging population, and bettering the health of middle-aged and senior citizens everywhere. This is the first research of its kind to investigate the correlation between Internet use and persons in their middle and later years; it provides new empirical evidence to existing studies. Furthermore, to ascertain the overall influence of mobile Internet use on the health of individuals in their middle years and above, we used the PSM model to remove endogeneity due to sample selection bias.

Nevertheless, a few caveats apply to our investigation. To begin, we utilized data collected from cross-sections of the population; however, the health of those in their middle years and those in their later years is a dynamic process. Therefore, in the future, we aim to use multiyear sub-tracking data to mor the results suggest that the estimates are more robust after eliminating endogeneity, and that failure to disentangle sample selectivity bias would overestimate not only the facilitating effect of mobile Internet use on the self-assessed health impacts of middle-aged and older adults, but also the ameliorating effect of mobile Internet use on the chronic diseases of middle-aged and older adults. Second, the explanatory variable was using a mobile APP, which is a binary variable; however, the frequency of mobile APP use also has different impacts on the well-being of the population as a whole. For instance, appropriate use of mobile APPs can improve the pleasure and happiness of the population; however, excessive use of mobile Internet or addiction will negatively affect population health. Therefore, in the future, a more in-depth examination of how technological progress affects the health of a population necessitates a deeper breakdown of mobile Internet use.

## Conclusions

Researching how mobile Internet utilization affects the health of the seniors and the middle-aged is practically important for promoting China’s comprehensive “Health China Strategy,” actively promoting healthy aging, and realizing the modernization of the health care system within the context of the digital China strategy and Health China Strategy. We used models like ordered logit and PSM to experimentally examine the influence of mobile Internet use on the health of those in their middle years and seniors in China leveraging data from the 2020 CHARLS. Our results imply that mobile Internet use substantially affects the state of health of individuals in their middle years and later in life, with positive effects on subjective health and the prevalence of the chronic disease. As per the findings of the heterogeneity assessment, the impacts of Internet use were more pronounced on the health of people in the middle age range and beyond between ages 45 and 60 than those aged ≥ 60 and older. In addition, to get rid of the endogeneity that came from bias in sample selection, we applied the PSM model. The results suggest that the estimates are more robust after eliminating endogeneity, and that failure to disentangle sample selectivity bias would overestimate not only the facilitating effect of mobile Internet use on the self-assessed health impacts of middle-aged and older adults, but also the ameliorating effect of mobile Internet use on the chronic diseases of middle-aged and older adults. The results of the mechanistic analysis suggest that social engagement is an important mediating mechanism between mobile Internet use and the health of middle-aged and older adults. This implies that mobile Internet use increases opportunities for social participation among middle-aged and older adults, thereby improving their health.

## Data Availability

The data of 2020 China Health and Retirement Longitudinal Study (CHARLS) is publicly available at https://charls.charlsdata.com/pages/data/111/zh-cn.html accessed on 16 November 2023.
